# Epidemiology of *Plasmodium vivax* in Duffy negatives and Duffy positives from community and health centre collections in Ethiopia

**DOI:** 10.1186/s12936-024-04895-1

**Published:** 2024-03-14

**Authors:** Lauren Bradley, Delenasaw Yewhalaw, Elizabeth Hemming-Schroeder, Brook Jeang, Ming-Chieh Lee, Endalew Zemene, Teshome Degefa, Eugenia Lo, Christopher King, James Kazura, Guiyun Yan

**Affiliations:** 1 Department of Ecology and Evolutionary Biology, School of Biological Sciences, University of California Irvine, Irvine, CA 92697 USA; 2 Department of Medical Laboratory Sciences and Pathology, College of Health Sciences, Jimma University, 5195 Jimma, Ethiopia; 3 Tropical and Infectious Diseases Research Centre, Jimma University, Jimma, Ethiopia; 4 Department of Microbiology, Immunology, and Pathology, Colorado State University, Fort Collins, CO 80523 USA; 5 School of Medical Laboratory Sciences, Faculty of Health Sciences, Jimma University, Jimma, Ethiopia; 6 Department of Microbiology and Immunology, Drexel University, Philadelphia, PA 19104 USA; 7 Center for Global Health and Disease, Case Western Reserve University, Cleveland, OH 44106 USA; 8 Program in Public Health, College of Health Sciences, University of California at Irvine, Irvine, CA 92697 USA

**Keywords:** Malaria, Duffy blood group, *Plasmodium vivax*, qPCR, Gene copy number

## Abstract

**Background:**

Malaria remains a significant cause of morbidity and mortality in Ethiopia with an estimated 3.8 million cases in 2021 and 61% of the population living in areas at risk of malaria transmission. Throughout the country *Plasmodium vivax* and *Plasmodium falciparum* are co-endemic, and Duffy expression is highly heterogeneous. The public health significance of Duffy negativity in relation to *P. vivax* malaria in Ethiopia, however, remains unclear. This study seeks to explore the prevalence and rates of *P. vivax* malaria infection across Duffy phenotypes in clinical and community settings.

**Methods:**

A total of 9580 and 4667 subjects from community and health facilities from a malaria endemic site and an epidemic-prone site in western Ethiopia were enrolled and examined for *P. vivax* infection and Duffy expression from February 2018 to April 2021. Association between Duffy expression, *P. vivax* and *P. falciparum* infections were examined for samples collected from asymptomatic community volunteers and symptomatic subjects from health centres.

**Results:**

Infection rate of *P. vivax* among Duffy positives was 2–22 fold higher than Duffy negatives in asymptomatic volunteers from the community. Parasite positivity rate was 10–50 fold higher in Duffy positives than Duffy negatives among samples collected from febrile patients attending health centres and mixed *P. vivax* and *P. falciparum* infections were significantly more common than *P. vivax* mono infections among Duffy negative individuals. *Plasmodium vivax* parasitaemia measured by 18sRNA parasite gene copy number was similar between Duffy positives and Duffy negatives.

**Conclusions:**

Duffy negativity does not offer complete protection against infection by *P. vivax*, and cases of *P. vivax* in Duffy negatives are widespread in Ethiopia, being found in asymptomatic volunteers from communities and in febrile patients from health centres. These findings offer evidence for consideration when developing control and intervention strategies in areas of endemic *P. vivax* and Duffy heterogeneity.

## Background

In spite of significant progress towards malaria control in the past two decades, malaria remains a major cause of mortality and morbidity in Africa [1]. According to the World Health Organization, *Plasmodium vivax* and *Plasmodium falciparum* contributed to approximately 700 thousand and 230 million cases, respectively, in Africa in 2021 [2]. In Ethiopia, there were an estimated 3.8 million cases in 2021 and 61% of the population resides in areas with endemic transmission [2, 3]. *Plasmodium vivax* and *P. falciparum* account for approximately 33% and 67% of all malaria cases, respectively, and it is one of only a few countries in Africa, where *P. vivax* remains consistently endemic [4].

Current endemicity of *P. vivax* in Africa correlates with areas of high heterogeneity in Duffy expression [4, 5]. The Duffy antigen receptor for chemokines (DARC), often referred to as the Fy glycoprotein, is a silent heptahelical chemokine receptor located on chromosome 1 and expressed on the surface of erythrocytes. DARC has been recognized as the binding antigen of *P. vivax,* and a single point mutation located in the GATA-1 transcription factor binding site of the DARC gene promoter (− 67 T > C) causes this receptor to not be expressed, resulting in a Duffy negative phenotype [6, 7]. The absence of this receptor on red blood cells has been shown to confer resistance to blood-stage infection by *P. vivax* [5, 8, 9]. This negative phenotype is nearly fixed in sub-Saharan Africa, correlating with the general lack of endemic *P. vivax* on the continent.

Despite this established dogma, cases of *P. vivax* are being found in confirmed Duffy negative individuals throughout different African countries [10–14]. In addition to Duffy blood group, other population-level factors that influence *P. vivax* epidemiology include climatic conditions, age, socioeconomic status, access to healthcare, malaria control measures [15, 16]. Whether discovery of increasing number of *P. vivax* infections in Duffy negatives results from more recent research on *P. vivax* in Africa or from new *P. vivax* genetic variants, the data suggest that Duffy negativity no longer confers complete resistance to blood-stage *P. vivax* infection [17, 18]. Furthermore, Duffy negatives generally develop reduced natural immunity to *P. vivax* blood-stage antigens [19–21].

There remains little information on the public health significance of *P. vivax* infection in individuals lacking the Duffy antigen in Africa. For example, how frequent are Duffy negative individuals infected with *P. vivax* compared to Duffy positive individuals from the same communities? How frequently does *P. vivax* contribute to clinical malaria among Duffy negatives compared to Duffy positives from areas of same endemicities? This population-based study aimed to address these questions in two locations with varying malaria endemicities in southwestern Ethiopia, using samples from communities and health centres.

## Methods

### Study sites

**Fig. 1:**
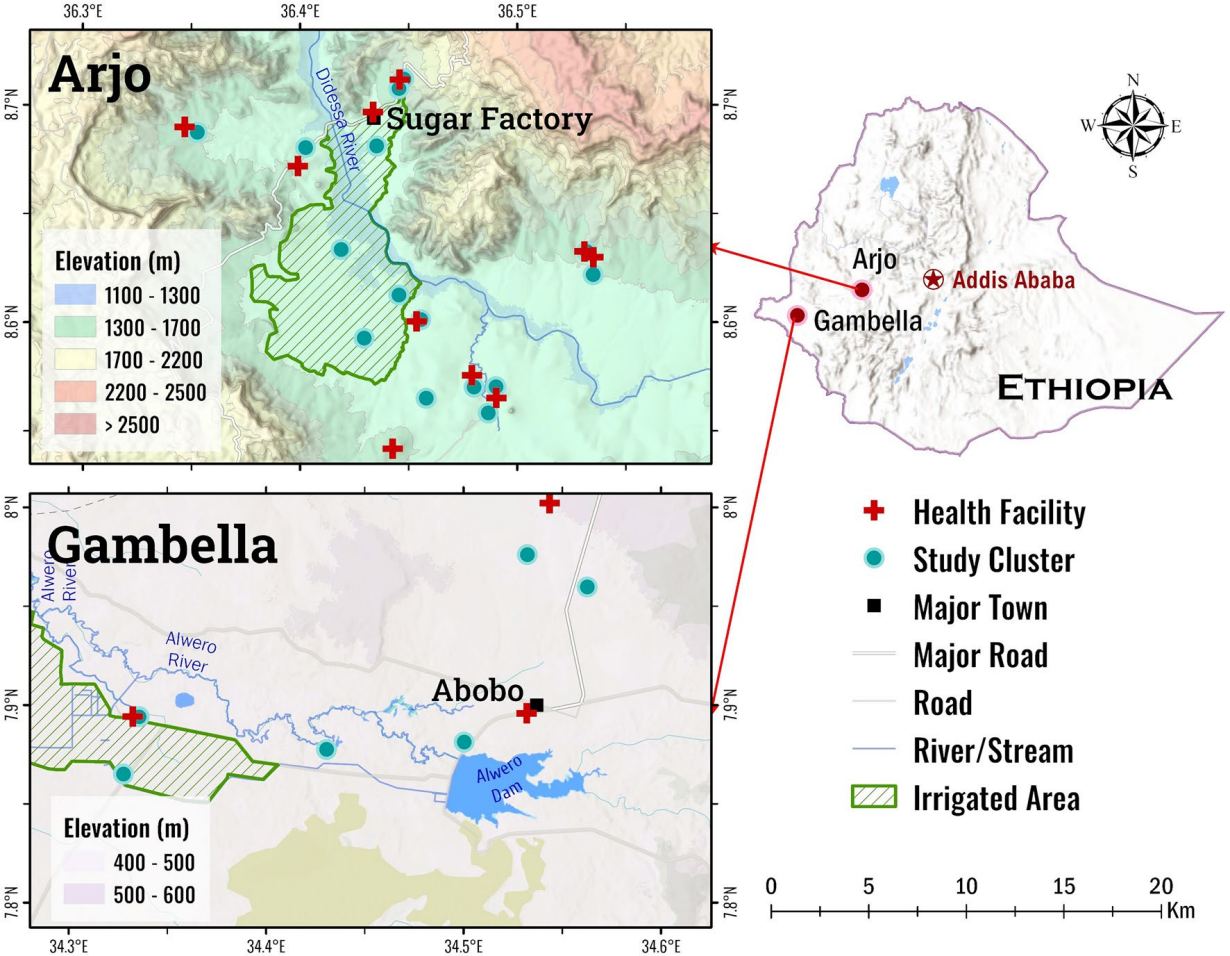
Map showing location of both study sites; Arjo and Gambella, in western Ethiopia. Includes locations of study clusters, health facilities, and major towns in the regions. The map was created with Esri ArcGIS Pro 3.1 with data sources from field survey, and elevation data from NASA SRTM v3 (https://doi.org/10.5067/MEaSUREs/SRTM/SRTMGL1.003)

Samples were collected from two study sites, Arjo-Didessa and Gambella, both located in western Ethiopia (Fig. 1) with a rainy season lasting from May to October. The Arjo-Didessa sugarcane plantation is located within the Oromia Regional State 395 km west of the Ethiopian capital Addis Ababa and the area covers most of the Arjo-Didessa sugarcane irrigation scheme. It is located at an elevation ranging from 1200 to 1500 m above sea level, and comprises 15 villages in 3 districts (Jimma Arjo, Bedele District, and Dado Hana District). It contains 1 health centre, 3 health posts, and 9 command posts which are smaller scale health posts located within the temporary residential areas formed by migrant workers. The sugarcane plantation was formerly the Didessa Wildlife Sanctuary before 2006 when the state owned sugarcane plantation was developed to supply the proximal sugarcane factory. It is one of the biggest sugarcane developments in the country, currently covering 5000 hectares with plans to expand to 80,000 hectares [22–24]. Gambella is located in the Abobo District in the Gambella Regional State, 811 km west of Addis Ababa. The area’s elevation ranges from 400 to 600 m above sea level and as of 2019 had a population of 20,080. The main socio-economic activity in the area is farming of cotton, maize and sorghum, or working fruit plantations to produce mango, papaya and banana. Additionally, the Alwero Dam provides fishing opportunities and employs approximately 2000 people at a large-scale rice irrigation scheme that currently spans 3000 hectares with plans to expand to 10,000 hectares. The district comprises 19 villages containing 4 health centres and 16 health posts [25, 26]. The populations at both locations primarily consisted of local villagers and migrant workers with long-term residency. These sites were chosen for the study as both areas have high levels of Duffy admixture, and continuous *P. vivax* endemicity [12, 27].

### Blood sample collection

Finger prick blood samples were collected throughout both study sites from community members who were asymptomatic during cross-sectional surveys, and from febrile volunteers attending health centres from February 2018 to April 2021. From each individual, a total of 3 blood spots, equaling ~ 50 ul, was pressed to Whatman 3MM filter paper for storage and transportation. For community collections all residents willing to participate were included in the study and provided signed informed consent and/or assent for minors under 18 years old. At the time of sample collection, for both clinical and community samples, the age and sex of participants were recorded when possible. Dried blood spots were transported to the University of California Irvine and stored at 4 °C.

### DNA extraction and qPCR of *Plasmodium* species

Parasite DNA was extracted from dried blood spots (DBS) using a standardized saponin/chelex method [28]. DNA was eluted to ~ 200ul molecular grade water stored at 4 °C in the short term or − 20 °C for long term storage. *Plasmodium* species-specific primers and probes were used to amplify the 18sRNA gene using a previously described protocol with modification [29]. Real time PCR was conducted at a total volume of 12ul containing; 6 µl ThermoFisher FastAdvanced MM (2X), 0.5 µl of each species-specific probe, 0.4 µl of each forward and reverse species-specific primer, and 2 µl parasite DNA. Reaction conditions were set as follows: 50 °C for 2 min, and 45 cycles of 95 °C for 2 min, 95 °C for 3 s, 60 °C for 30 s and run on a QuantStudio 3 Real-Time PCR System.

### Duffy sequencing

An approximately ~ 600-bp fragment of the human DARC gene encompassing the − 33rd nucleotide position located in the GATA-1 box of the promoter region was amplified sequenced following established protocols to assess Duffy expression [27, 30, 31]. Specifically, the total volume for amplification was a 20ul reaction mixture containing; 10 µl DreamTaq Green PCR MM (2X), 0.3 µl of each forward and reverse primer, and 2 µl genomic DNA. Thermocycling conditions were set at; 94 °C for 2 min, 35 cycles of 94 °C for 30 s, 61 for 30 s, and 65 for 40 s followed by a 2-min extension at 65 °C. Five microliters of PCR product were run on a 1.5% agarose gel to confirm amplification. PCR product which had successful amplification was cleaned enzymatically to remove remaining primers and dNTPs; 2 µl SAP and 0.2 µl XO1 per was added to PCR product and cleaned via the following thermocycling conditions; 37 °C for 15 min, 80 °C for 15 min, and then held at a 4C extension. Sanger sequencing was conducted by Retrogen Inc. using forward primers and chromatogram results were visually analysed via Chromas for a T → C mutation at the 33rd nucleotide position indicating Duffy negativity. Only samples positive for *P. vivax* mono and mixed infections were sequenced for Duffy expression.

### Data analysis

Malaria prevalence was calculated for both study settings at each study site separately. Overall prevalence of both *Plasmodium* species was compared between study sites for community and clinical collections via the Chi-Square test for independence. Given that only *P. vivax* positive samples were sequenced for Duffy expression, rates of Duffy negativity in the population was not directly assessed for this study, the overall rate of *P. vivax* in Duffy negative and Duffy positive individuals was calculated by dividing the number of *P. vivax* infections by the expected number of Duffy negative and positive individuals at each site. Expected Duffy negative and positive populations were calculated by multiplying the total number of samples by the Duffy negativity rate in Arjo (43.6%) and Gambella (45.9%) as determined in a previously published study [21]. The ratio of mixed (*P. vivax* + *P. falciparum*) to mono (*P. vivax*) infections was determined for each study setting, community and health facility, for both Duffy negatives and Duffy positives.

Comparisons of the rate of *P. vivax* in Duffy negatives to Duffy positives, and the ratio of mixed to mono infections for Duffy negatives and positives were made via Fisher’s Exact test for both community and health facility collected samples. Parasite Gene Copy Number (GCN) was calculated from qPCR Cycle threshold (Ct) values via standard curve to estimate parasite density. Log_10_ transformed GCN was compared between community and health facility settings for both *P. vivax* and *P. falciparum* via two-sample t-test, and between Duffy negatives and Duffy positives for both settings via Fisher’s Exact test.

## Results

### Prevalence of *P. vivax* across study sites, collection method and Duffy expression

A total of 14,247 dried blood spots were collected from two study sites in southwestern Ethiopia (Fig. 1) from February 2018 to December 2021. Asymptomatic community collections were made via cross-sectional surveys conducted during the spring and late-fall of each year and making up 9580 of the total dried blood spots. The remaining 4667 samples were from symptomatic infections collected from health clinics and facilities in the regions via passive case detection (PCD). In total 344 DBS were positive for only *P. vivax*, 937 for only *P. falciparum* and 35 samples exhibited a mixed infection being positive for both *P. vivax* and *P. falciparum* (Table 1). A total of 7519 of these DBS were collected from Arjo; 5454 from cross-sectional surveys and 2065 from passive case detection. In Gambella 6728 samples were collected in total; 4126 were collected from the community during cross-sectional surveys and 2602 via passive case detection (Table 1). Overall, *P. vivax* and *P. falciparum* infection rate was significantly higher in Gambella than in Arjo (P < 0.001 for both species).

**Table 1:** PCR Prevalence of *Plasmodium vivax* (Pv) and *P. falciparum* (Pf) infections among community-based asymptomatic sampling and sample positivity among febrile patients detected by passive case surveillance from health centres in two sites in Ethiopia

Settings	Site	Samples (n)	Total *Plasmodium* infections	Mixed Pv and Pf infections	Pv mono infections	Pf mono infections	P-value*
Community	Arjo	5454	19 (0.35%)	0	3 (0.06%)	16 (0.29%)	< 0.05
	Gambella	4126	424 (10.28%)	8 (0.19%)	133 (3.22%)	283 (6.86%)	< 0.001
	Total	9580	443 (4.62%)	8 (0.08%)	136 (1.42%)	299 (3.12%)	
Health Centre	Arjo	2065	313 (15.16%)	9 (4.36%)	114 (5.52%)	190 (9.20%)	< 0.001
	Gambella	2602	560 (21.52%)	18 (0.69%)	94 (3.61%)	448 (17.22%)	< 0.001
	Total	4667	873 (18.79%)	27 (0.57%)	208 (4.46%)	638 (13.67%)	

^*^Fishers exact test comparison between Pv and Pf mono infection rate

Duffy genotyping was performed only on *P. vivax* mono infections and mixed *P. vivax* and *P. falciparum* infections across all study sites and collection methods (Table 2). Of the 379 *P. vivax* positive and mixed-species infections, 345 were successfully sequenced at the T33C promoter of the GATA-1 transcription factor. Among the community-based cross-sectional samples, infection rate of *P. vivax* among the Duffy negatives and positives was low and similar in Arjo, but significantly higher infection rate was found in Gambella among Duffy positives than Duffy negatives (5.6% vs. 0.26%, P < 0.001; Table 2). Similarly, sample positivity rate was more than 10–50 fold higher in Duffy positive than Duffy negatives in both sites among samples collected from the health centre settings (Table 2), suggesting a much reduced *P. vivax* burden among Duffy negative people in febrile patients.

**Table 2:** Rate of *Plasmodium vivax* (Pv) infections among Duffy negative and Duffy positive individuals in both community-based asymptomatic and passive case surveillance from health centres at two study sites in Ethiopia

Setting	Site	Duffy negatives	Duffy positives	Rate of Pv in Duffy negatives	Rate of Pv in Duffy positives	P-value*
Community	Arjo	2525	2929	0.04% (1/2525)	0.07% (2/2929)	> 0.05
	Gambella	1894	2232	0.26% (5/1894)	5.60% (125/2232)	< 0.001
Health Centre	Arjo	956	1109	0.21% (2/956)	10.64% (118/1109)	< 0.001
	Gambella	1194	1408	0.59% (7/1194)	6.04% (85/1408)	< 0.001

^*^P-value calculated via Fishers Exact Test for comparing Rate of Pv in Duffy Negatives to Duffy Positives

Interestingly, a considerably large proportion of malaria infections were mixed species infection. Among the 133 successfully sequenced community-based samples, eight out of 133 (6.0%) malaria infections were mixed species and 26 out of 212 (12.3%) samples were mixed infections from the health centre settings (Table 3). Among the Duffy negatives, *P. vivax* was found more frequently found in the form of mixed-species infection than mono infections, whereas mono *P. vivax* infections were far more common in Duffy positives. In the community asymptomatic samples, the ratio of mixed species infection to *P. vivax* mono infection was 0.5 among Duffy negatives, but this ratio was reduced to 0.05 in Duffy positives (P < 0.05; Table 3). In febrile samples from health centres the ratio of mixed species infection to *P. vivax* mono infection was 3.5 among Duffy negatives, far greater than the ratio observed in Duffy positive (0.10; P < 0.001). This data strongly suggests that in Duffy negative individuals *P. vivax* is more frequently found in mixed infections compared to *P. vivax* only mono infections.

**Table 3:** Distribution of Duffy phenotypes across *Plasmodium vivax* (Pv) and Mixed (Pv and Pf) infections in both community-based asymptomatic and passive case surveillance via health centers from two study sites in Ethiopia

Setting	Infection	n	Duffy negative	Duffy positive	Ratio of mixed species infection to *P. vivax* only infections		Fisher’s exact test
					Duffy negative	Duffy positive	
Community	Pv	125	4	121	0.5	0.05	P < 0.05
	Pv + Pf	8	2	6			
Health Center	Pv	186	2	184	3.5	0.10	P < 0.001
	Pv + Pf	26	7	19			

### *Plasmodium vivax* parasitaemia in community and clinical samples and across Duffy expressions

**Fig. 2:**
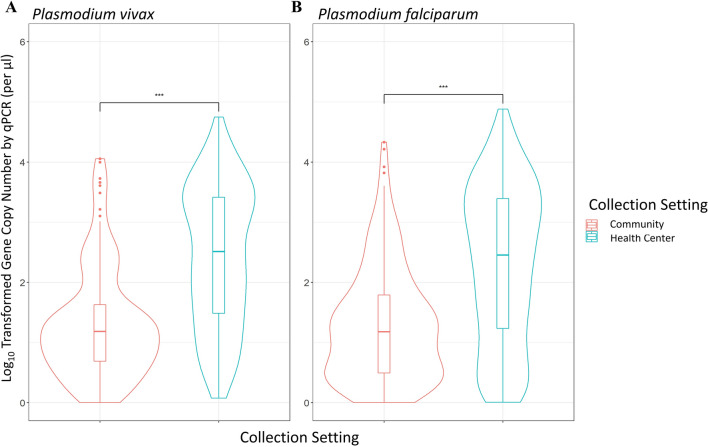
Violin plots of the log-transformed malaria parasite gene copy number of samples collected from asymptomatic communities and febrile patients of all ages from health centres in Ethiopia. **A** *Plasmodium vivax,* and **B** *P. falciparum* by qPCR for individuals of all ages. The central box represents the interquartile range with the median shown as the centre line in the box. ***, P < 0.001 based on Fisher’s exact test

**Fig. 3:**
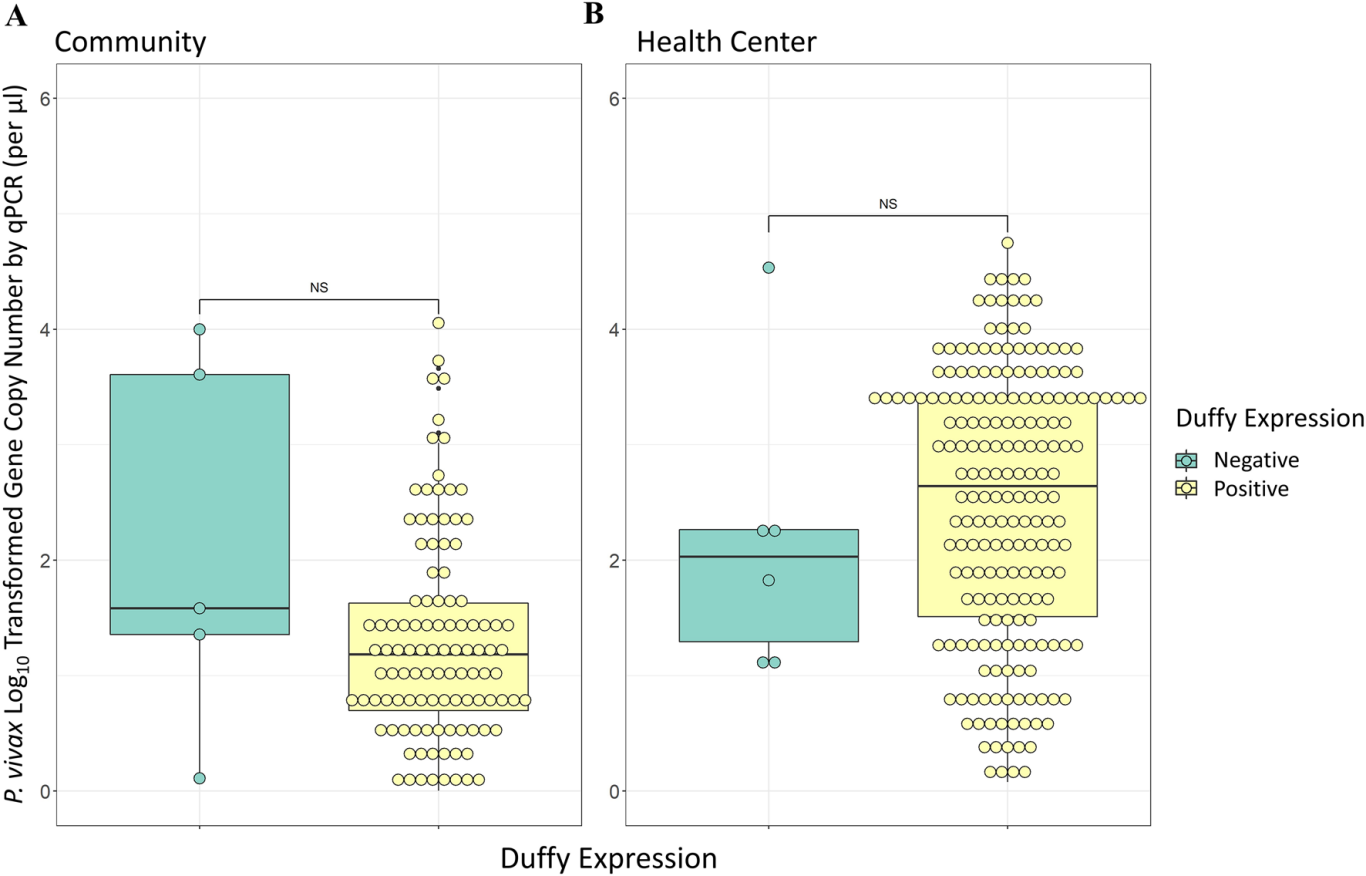
Box plots of the log-transformed *Plasmodium vivax* parasite gene copy number for Duffy negative and Duffy positive individuals of all ages. **A** asymptomatic community samples; and **B** febrile malaria samples from health centres. Box plots represent the interquartile range with the median expressed as the centre line. NS, non-significant based on Fisher’s exact test

Analyses of qPCR data revealed significant differences in the parasitaemia between cross-sectional samples without clinical symptoms and clinical samples collected during passive case detection from health centres for both *P. vivax* and *P. falciparum*. In both *P. vivax* and *P. falciparum* infections parasitaemia was significantly higher in samples collected via passive case detection than via cross-sectional survey (P < 0.001, Fig. 2). Symptomatic *P. vivax* infections showed a geometric mean gene copy number (GCN) of 2.03 parasites/µl, which was significantly higher than the asymptomatic *P. vivax* infections, which had a geometric mean of 0.94 parasites/µl (P < 0.001, Fig. 2). Similarly, symptomatic *P. falciparum* infections exhibited a geometric mean of 1.67 parasites/µl, which was significantly higher than the asymptomatic *P. falciparum* infections which had a mean of 0.90 parasites/µl (P < 0.001). Community Duffy-negative and Duffy-positive samples exhibited a similar parasitaemia, with a GCN of 1.28 and 0.93 parasites/µl, respectively (P > 0.05, Fig. 3). Similarly, PCD Duffy-negative and Duffy positive samples showed a mean GCN of 1.93 and 2.07 parasites/µl, respectively (P > 0.05, Fig. 3). These data do not include four Duffy negative samples as their gene copy numbers fell just outside of our standard curve based cut-off range. Given the substantial differences in sample sizes between Duffy-negatives and Duffy-positives it is possible that the lack of significance observed here is indeed due to a small samples size of Duffy negatives.

## Discussion

This study sought to examine *P. vivax* malaria burden in Duffy negative individuals at two field sites with similar proportion of Duffy negativity, but different malaria endemicities in southwest Ethiopia. *Plasmodium vivax* posed a significant health burden at both sites, but was far more prevalent in the community in Gambella than in Arjo where infection prevalence was over 50 times higher. In febrile patients *P. vivax* was found more often in Arjo than in Gambella; however, this difference was much less drastic than in the community with Arjo exhibiting only 1.5 times more *P. vivax* clinical infections than Gambella. Across both sites and collection settings *P. vivax* was found far less frequently in Duffy negatives than Duffy positives. In the community Duffy positives had approximately 2 and 22-fold greater infection rate of *P. vivax* than Duffy negatives at Arjo and Gambella, respectively. In febrile patients and samples collected from health facilities this trend was even more apparent; in Arjo and Gambella Duffy positives exhibited a 51 and tenfold greater positivity rate of *P. vivax* infections, respectively, than Duffy negatives. The variations in rate of infection were highly significant for samples from health centres at both sites, but only significant for community samples from Gambella. The lack of significance in Arjo community samples could potentially be due to the small sample size as only three *P. vivax* infections were found in the community in Arjo. These strongly suggest that *P. vivax* infections, though commonly found in Duffy negative individuals, are still predominantly occurring in Duffy positive people. Despite the significant variations in rate of *P. vivax* infection between Duffy expressions, significant differences in parasitaemia between Duffy negatives and Duffy positives was not observed in either the community or health centres. Perhaps most interestingly this study highlights a pattern of mixed versus mono infections related to Duffy negativity. The ratio of mixed to mono *P. vivax* infections among Duffy negatives exhibited a 10 and 35-fold greater ratio than Duffy positives in both the community and clinical settings, respectively. Therefore, for Duffy negatives, *P. vivax* is predominantly found in mixed infections more than mono infections.

Since the level of *P. vivax* exposure remained consistent among both Duffy positive and Duffy negative individuals across both study locations, the observed diminished burden of *P. vivax* in Duffy negative individuals underscores that while Duffy negativity does not confer absolute resistance to *P. vivax* infection, it does exert a significant inhibitory effect on infection establishment. The mechanism behind *P. vivax* infections of Duffy negatives remains highly elusive, however, several studies have highlighted potential invasion mechanism adaptations of *P. vivax* that may circumvent Duffy-based infection inhibition and allow for infection on a lesser scale [17, 32]. One of the most well studied of these potential adaptations is the *P. vivax* Duffy binding protein 1 (PvDBPI) copy number expansion. Several different studies have clearly shown that PvDBP gene amplification both facilitated binding to alternative lower affinity receptors in Duffy negatives, and also suggested that the binding affinity of DARC with high copies of PvDBP could be much higher than with single-copy PvDBP parasites [17, 33–35], providing a potential selective pressure towards gene duplication and thus increased infectivity. Two additional ligands, *P. vivax* glycosylphosphatidylinositol-anchored micronemal antigen (PvGAMA) and *P. vivax* merozoite surface protein-1 paralog (PvMSP1P), were recently found capable of binding to both Duffy positive and negative red blood cells, suggesting possible involvement in Duffy-independent invasion pathways [36].

Collectively these findings build on previous work documenting *P. vivax* infections in Duffy negative individuals in numerous African countries [37, 38] including Cameroon [39], Madagascar [10], Angola and Equatorial Guinea [40], Kenya [41], Ethiopia [4]. These studies are consistent with the current findings and support the conclusion that Duffy negative individuals are not completely resistant to infection by *P. vivax*, yet still have a greatly reduced prevalence of *P. vivax* infections compared to Duffy positive individuals. This data shows that regardless of exhibiting no significant variation in parasitaemia between Duffy positives and Duffy negatives, several *P. vivax* infections from Duffy negatives exhibited relatively high levels of parasitaemia potentially implying that these parasites readily infect and adapt to Duffy negativity, allowing for greater erythrocyte invasion. Despite this, several studies have ample evidence that parasitaemia of *P. vivax* is greatly reduced in Duffy negatives, supporting the hypothesis that parasite infectivity to the human erythrocyte, though not completely inhibited, is indeed reduced in the absence of the Duffy antigen [42, 43]. Several prior studies have also observed that *P. vivax* infections within Duffy negative individuals are frequently mixed infections, yet these data are limited in that they are predominantly descriptive and do not explore these mixed infections in detail nor compare their prevalence between Duffy negatives and positives [10, 44, 45]. Thus this current study stands out in its efforts to systematically evaluate the prevalence of mixed infections in individuals with Duffy negative status as compared to those with Duffy positive status. These findings thus shed light on the noteworthy phenomenon that *P. vivax* infections in Duffy negatives frequently encompass mixed-species infections, especially when compared to Duffy positives.

It warrants mention that in the present study is limited in that Duffy expression (negative vs. positive) was inferred based on genotype data of the T33C point mutation in the promoter region of the GATA-1 transcription factor binding site of the Duffy antigen receptor for chemokines (DARC) gene, which is known to alter erythroid expression and eliminate Duffy antigen expression on the red blood cell surface [30, 31, 46]. However, the direct antigen expression (phenotype) was not assessed. It is, therefore, possible for a genotypically categorized Duffy negative individual to potentially express Duffy receptors in some quantity, and the *P. vivax* strains infecting Duffy negatives in this study may be utilizing such an expression in invasion, despite genotypic negativity. Additionally, neither prevalence nor burden of *P. falciparum* across Duffy negatives and positives as Duffy expression is not known to be associated with *P. falciparum* infection.

## Conclusion

Understanding the distribution of *P. vivax* in Africa and exploring the significance of Duffy expression continues to be a challenging and intricate endeavour. Given the low parasitaemia often associated with *P. vivax* infections of Duffy negative individuals, microscopy and RDTs are often not sensitive enough to detect infection, hindering their diagnosis and study in the field. Indeed, corresponding microscopy data from this area accounted for only approximately 70%, of all qPCR confirmed *P. vivax* positive infections [22], highlighting the need for more sensitive molecular detection tools in the field. This has significant implications for malaria elimination on the continent as a high proportion of *P. vivax* cases are likely being overlooked by traditional diagnostic methods. This work shows that not only does *P. vivax* transmission remain widespread in Ethiopia, but these asymptomatic community infections make up a significant portion of *P. vivax* cases resulting in a large undetected parasite reservoir that may greatly complicate and hinder interventions and elimination efforts. Finally, it is clear through the current study that Duffy negativity is not a definitive barrier to infection, and *P. vivax* infections were detected in Duffy negative individuals, encompassing both asymptomatic and febrile malaria instances, frequently occurring in mixed infections. Importantly, these trends persist across both study sites representing high and low endemic settings. This information is vital to informing control and elimination strategies in areas of sub-Saharan Africa with variable *P. vivax* endemicity and of high Duffy heterogeneity.

## Data Availability

The datasets used and/or analyzed during the current study are available from the corresponding author on reasonable request.
